# Unique Sensitivity of Uterine Tissue and the Immune System for Endometriotic Lesion Formation

**DOI:** 10.3389/fphys.2021.805784

**Published:** 2021-12-17

**Authors:** Stephanie A. Morris, Kenneth S. Korach, Katherine A. Burns

**Affiliations:** ^1^Department of Environmental Health, Division of Environmental Genetics and Molecular Toxicology, University of Cincinnati College of Medicine, Cincinnati, OH, United States; ^2^Receptor Biology Group, Reproductive, and Developmental Biology Laboratory, National Institute of Environmental Health Sciences (NIEHS), National Institutes of Health, Research Triangle Park, NC, United States

**Keywords:** endometriosis, uterine tissue, immune system, matrix metalloproteinase, estrogen, MMP7

## Abstract

Endometriosis is a debilitating disease that affects about 10% of reproductive-aged adolescents and women. The etiology of the disease is unknown; however, a prevailing hypothesis is that endometriosis develops from retrograde menstruation, where endometrial tissue and fluids flow back through the oviducts into the peritoneal cavity. There is no cure for endometriosis, and symptoms are treated palliatively. Despite the advances in knowledge, the complexity of endometriosis etiology is still unknown. Recent work by our group suggests that the initiation of endometriosis is immune-dependent. Using a mouse model of endometriosis, we hypothesized the initiation of endometriosis is immune regulated and uterine endometrium specific. In the absence of a functional immune system non-obese diabetic/severe combined immunodeficiency (NOD/SCID mice), endometriotic lesions did not form. Uterine endometrial tissue forms endometriotic lesions, whereas tissues with differing basal expression levels of estrogen receptor alpha (ESR1) and estrogen receptor beta (ESR2), similar cellular composition to uterus (i.e. bladder, mammary gland, and lung), and treated with estradiol did not form lesions. As MMP7 is known to play a major role in the organization/reorganization of the endometrium during the menstrual cycle, blocking metalloproteinase (MMP) activity significantly decreased the invasive properties of these cells. Together, these findings suggest that endometriosis is immune and uterine specific and that MMP7 likely plays a role in the ability of uterine tissue and the innate immune system to establish and maintain endometriotic lesions.

## Introduction

Endometriosis affects about 10% of reproductive-aged adolescents and women with debilitating disease symptoms that include painful menstruation, chronic pain, pain with intercourse, and infertility (Giudice and Kao, 2004; Rogers et al., 2009). A leading theory for the development of endometriosis is *via* retrograde menstruation, where viable endometrial tissue flows back through the oviduct and into the peritoneal cavity. In the peritoneal cavity, the uterine endometrial tissue attaches to multiple foreign sites (e.g., fallopian tubes, ovarian fossa, peritoneal wall, ligaments, and bowel) (Sampson, 1927; Syrop and Halme, 1987). Despite the advances made in the study of endometriosis, the complex etiology is still largely unknown.

Endometriosis is often described as an estrogen (E_2_)-dependent disease; however, we described that the initiation of endometriosis is immune-mediated with the progression of endometriosis being hormone-mediated (Burns et al., 2012, 2017). These findings correspond with what is known in women with endometriosis because hormone-dependent and/or responsive treatments are not curative, and upon cessation, symptom recurrence is about 70% (Giudice and Kao, 2004; Selcuk and Bozdag, 2013). Throughout the body, estrogens exert their effects through ESR1 and ESR2 in multiple organ systems, but estrogen is also both pro- and anti-inflammatory (Capellino et al., 2006).

Women with endometriosis have higher incidences of autoimmune disorders (Bruner-Tran et al., 2002a; Kyama et al., 2009; Ricci et al., 2011). Their peritoneal fluid has an elevated number of activated macrophages (Halme et al., 1987; Sharpe-Timms, 2001; Bulun et al., 2002; Khan et al., 2004; Capellino et al., 2006; Bukulmez et al., 2008). Cytokines produced from the cells of the innate immune system are critical for mediating cellular recruitment, neoangiogenesis, and resolution of inflammation (Fantin et al., 2010; Mantovani et al., 2011; Phillipson and Kubes, 2011; Sadik et al., 2011; Christoffersson et al., 2012; Kolaczkowska and Kubes, 2013). Among these cells, neutrophils are major effectors of acute and chronic inflammatory conditions, whereas macrophages are involved in the repair processes (Murray and Wynn, 2011; Kolaczkowska and Kubes, 2013). Our previous study suggested that neutrophils and macrophages mediate the initiation of endometriotic lesion formation (Burns et al., 2017). Multiple immune-compromised mice exist which allows for the study of specific aspects of immune regulation. The non-obese diabetic (NOD) mice have autoimmune characteristics with autoimmune CD4/CD8^+^ T cells, and they lack the MHC haplotype H2^G7^ (Kikutani and Makino, 1992). The SCID mice have nonfunctional B/T cells and have leaky circulation levels of immunoglobulin M (IgM), immunoglobulin G (IgG), or immunoglobulin A (IgA) (Bosma and Carroll, 1991). In combination, the NOD/SCID mice exhibit natural killer dysfunction, low cytokine production, B/T cell dysfunction, absence of complement, and defective macrophage and dendritic cells, which leads to a severely immunocompromised mouse line (Shultz et al., 1995).

Based on our previous studies, we hypothesized that uterine endometrium and the immune system are necessary for the development of endometriotic lesions. To test our hypothesis, we used an endometriosis mouse model in which change to: 1) NOD/SCID mice were used as both host and donor for uterine endometrium to establish endometriotic lesions in a hormonally intact mouse and (2) wild-type (WT) donor tissues with different basal levels of expression of ESR1 and ESR2 and similar cellular composition to the uterus (i.e., uterine, lung, bladder, and mammary) were used to establish endometriotic lesions in ovariectomized mice with supplemental E_2_. In line with our hypothesis, we found that endometriotic lesions, defined as having glands, stroma, organized structure, hemosiderin-laden macrophages, and fibrosis, do not form in the severely immunocompromised mice. Additionally, suggesting a strong uterine dependence, regardless of ESR1 and ESR2 status, only the uterine tissue dispersed into the peritoneal cavity forms lesions. Together, our findings provide further evidence that the initiation of endometriosis is immune-mediated and uterine-specific, and lesion growth progression is driven by estrogen.

## Materials and Methods

### Animal Care and Treatment

All animal studies were conducted in accordance with the National Institutes of Health Guidelines for Humane Use and Care of Animals and the Institutional Animal Care and Use Committee (IACUC) of the University of Cincinnati and followed the Panel on Euthanasia of the American Veterinary Medical Association. Transgenic C57BL/6-Tg (UBC-GFP) 30Scha/J (GFP) (IMSR Cat# JAX:004353, RRID: IMSR_JAX:004353) sexually mature breeders, 6 to 8-week-old WT C57BL/6J (IMSR Cat# JAX:000664, RRID: IMSR_JAX:000664), and NOD.CB17-*Prkdc*^*scid*^/J (NOD/SCID) (ISMR Cat# JAX:001303, RRID: IMSR_JAX:001303) female mice were purchased from Jackson Laboratories. Female mice aged between 2 and 4 months were used. Mice were in a controlled temperature range (72–74 F) on a 12 h light/12 h dark cycle. They were given food and water *ad libitum*. For example, groups were designated in the following manner (donor to host): WT to WT, NOD/SCID to NOD/SCID.

Endometriosis was induced according to our established protocol (Burns et al., 2012, 2017) in hormonally intact mice. Specifically, to study the role of the immune system in endometriosis, intact NOD/SCID and WT mice were used. Endometriosis was initiated randomly, and the surgeons were blinded as to mouse genotype. Briefly, all donor mice, GFP and NOD/SCID, were primed 41 h prior to uterus removal with pregnant mare serum gonadotropin (PMSG) 3.5 international units (IU) intraperitoneally (Burns et al., 2012). The donor uterus was removed *en bloc* after euthanasia and placed into sterile phosphate-buffered saline (PBS). The outer myometrium of the uterus was gently peeled away from the endometrium, the uterus was splayed open, and minced (≤ 1.5 mm). Host mice were anesthetized using isoflurane/oxygen and given buprenorphine (0.1 mg/kg) for pain management. A 0.5 cm right dorsolateral incision was made in the host abdomen, while the donor endometrial uterine tissue was minced. The minced donor uterine tissue (~50 mg) was suspended in 500 μl of PBS and dispersed into the peritoneal cavity of the host, the peritoneal wall was overlapped to close the cavity, and the outer skin was closed with 9 mm clips (*n* = 8 per group). Endometriotic lesions were allowed to establish for up to 3 weeks, and all mice were euthanized in the estrus phase of the estrous cycle. Estrous cycle progression was performed daily one week prior to harvest *via* vaginal lavage and microscopic analysis as previously described (Jayes et al., 2014). Vaginal cytology was read and confirmed blindly by at least two people. All necropsies were blinded to the person collecting the lesions.

To determine the role of morphologically similar tissues, which express different basal levels of ESR1 and ESR2, and the role of estradiol in the attachment of tissues, host C57BL/6J mice were ovariectomized through two 0.5 centimeter dorsolateral skin incisions and endogenous hormones were allowed to clear for 7-10 days. Mice were then randomly divided into two treatment groups, estradiol valerate (E_2_; IUPAC: [(8R,9S,13S,14S,17S)-3-hydroxy-13-methyl-6,7,8,9,11,12,14,15,16,17-decahydrocyclopenta[a]phenanthren-17-yl] pentanoate) (2.5 μg/mouse/week; Sigma-Aldrich, St. Louis, MO) in corn oil or corn oil vehicle (*n* = total of 6 mice per group). Mice were dosed subcutaneously once prior to experimental induction of endometriosis and then once weekly for 3 weeks. All donor GFP mice were primed 41 h prior to uterus removal with PMSG 3.5 IU intraperitoneally (Burns et al., 2012). The donor tissue, uterus, mammary gland, bladder, and lung were removed *en bloc* after euthanasia. Tissue samples were cleaned of any excess tissue and minced (<1.5 mm). Host mice were anesthetized as above, and tissue (~50 mg) was dispersed into the host abdomen as above. Endometriotic lesions were allowed to establish for up to 3 weeks. All necropsies were blinded to the person collecting the lesions.

After 3 weeks, mice were euthanized with CO_2_. Peritoneal lavage was performed by injecting 1 ml of PBS + 0.5% BSA + 2 mM EDTA into the peritoneal cavity. The cavity was gently massaged, a small incision was made into the inner skin lining the peritoneal cavity, and the fluid was gently removed without drawing organs into the syringe. The peritoneal lavage was used to obtain cells for cytospins for differentials (see below). Ectopic tissues were photographed to document *in situ* images of endometriotic lesions and/or if lesions were formed from the other tissues (Leica dissecting microscope M250FA and Leica camera DFC450C, Germany). Non-uterine tissue fragments were found to be unattached within the entire peritoneal cavity, these were pooled together on top of the intestine and documented. Endometriosis lesions and tissue fragments were visualized, dissected, measured, weighed and then removed and either fixed in 10% formalin or snap frozen on dry ice and stored at −80 °C until use.

### Cytology

Fixed tissues were routinely processed for paraffin embedding. Five-micron sections were cut, and the slides were used for H&E staining following standard protocols. All the slides were deparaffinized and hydrated through descending grades of alcohol, stained, dehydrated, and cover-slipped.

Differentials were stained using modified Giemsa (Easy III, Azer Scientific, Morgantown, PA) according to the protocol of the manufacturer. All slides were dehydrated and cover-slipped.

### RNA Isolation and Real-Time PCR

Frozen endometriosis lesions and tissues removed from the mice were pulverized under liquid nitrogen, and RNA was isolated using TRIzol as per manufacturer's instructions (Invitrogen, Carlsbad, CA, USA). Using a previously described method, cDNA was synthesized and analyzed by real-time PCR using Fast SYBR (ThermoFisher Waltham, MA, US) (Winuthayanon et al., 2009). Relative transcript levels were quantified in comparison with the WT to WT vehicle group and normalized to *Rpl7* using the model described by Pfaffl (2001). Primer sequences (Table 1) purchased from Fisher Scientific were selected using Primer Express (Applied Biosystems, Foster City, CA, USA), Harvard Primer Bank (Harvard University, Boston, MA, USA), or PrimerBot! (McDonnell Lab, Duke University, Durham, NC, USA).

**Table 1:** Primer sequences.

**Gene**	**Forward**	**Reverse**
*Rpl7*	5′-AGCCCAAAGGTTCGTAAGGT-3′	5′-CATGCAATGTATGGCTCCAC-3′
*Mmp2*	5′-CAAGTTCCCCGGCGATGTC-3′	5′-TTCTGGTCAAGGTCACCTGTC-3′
*Mmp3*	5′-ACATGGAGACTTTGTCCCTTTTG-3′	5′-TTGGCTGAGTGGTAGAGTCCC-3′
*Mmp7*	5′GGATGAGTACTGCTGGACTGATGGTGA-3′	5′-AGAGTGGCCAAATTCATGGG-3′
*Mmp8*	5′-ATTCCCAAGGAGTGTCCAAG-3′	5′-TCATATCTCCAGCACTGGTTG-3′
*Mmp9*	5′-GCGTGTACGGACCCGAAG-3′	5′-AGGGATACCCGTCTCCGTG-3′
*Mmp10*	5′-GGCCCACTCTTCCTTCAGAC-3′	5′-TTCATTTCCTCGGACTGCCC-3′
*Mmp12*	5′-TGGGCTTCTCTGCATCTGTG-3′	5′-CATGAGCTCCTGCCTCACAT-3′
*Mmp25*	5′-CTTTGATGCCGTTGCCAACA-3′	5′-CTTTCACATGGGTTGGCAGC-3′

### Cell Culture and Cellular Invasion

Mouse endometriosis lesion (mEmLe) cells were generated from mouse endometriotic lesions by removing the lesions *en bloc*, washing with sterile PBS, and placing them into DMEM. The lesions were cut in ~1 mm pieces and placed into a 6 well culture dish with enough DMEM + 10% FBS + antibiotic/antimycotic (penicillin, streptomycin, Amphotericin B, A5955, Sigma) to just cover the tissue pieces at 37°C and 5% CO_2_. The lesions were allowed to attach for 24-48 h. Once attached, the lesion pieces were left to allow the cells to migrate from the tissue onto the plate. Multiple lines were generated; however, the mEmLe cells were sorted for GFP positivity to ensure donor derived cells and the expression of genes associated with endometriotic lesions (Zeitvogel et al., 2001). The line is maintained in 1:1 conditioned media DMEM : DMEM + 10% FBS + antibiotic/antimycotic at 37°C and 5% CO_2_. For invasion assays, a ChemoTx® 48-well micro chemotaxis chamber (Neuroprobe, Gaithersburg, MD, USA) was used. In the lower chamber, 5% FBS in DMEM was used as the chemoattractant. Sandwiched between the chambers, growth factor-free Matrigel (0.5 mg/ml in 0.7% NaCl + 0.01 M Tris pH 8; Corning, Corning, NY, USA) was applied to an 8 μM micropore membrane before the cells were introduced to the well. For actinonin (AdipoGen, 13434-13-4, MFCD00080257) treatment, it was added to the media at setup or for pretreatment, the cells were pretreated with 50 μM actinonin for 16 h before being placed into the upper chamber. The chamber was incubated at 37°C and 5% CO_2_ for 18 h. The chamber was disassembled, and cells were removed from the non-migrated side of the membrane. The migrated cells were fixed to the membrane in methanol for 10 min. The membrane and cells were stained with 0.1% crystal violet + 0.1% methanol for 15 min, then dipped in distilled water 4 times. The membrane was mounted on a slide using Permount (Permount, Fisher Scientific, Fair Lawn, NJ, US). A comparison of densitometry was made using Image J software (National Institutes of Health, Bethesda, MD, US).

### Statistical Analysis

The one-tailed unpaired *t*-test using Welsh's correction, one-way ANOVA using Welsh's correction and Dunnett's T3 multiple-comparison test, and the two-way ANOVA using Tukey's multiple comparison test, *p* < 0.05, were performed using GraphPad Prism version 9 (GraphPad Software, San Diego, CA, USA). The standard error of the means not sharing a letter are significantly different from each other (*p* < 0.05). Standard error of the means sharing the same single letter or a letter in combination with other letters are not significantly different from each other (*p* > 0.05).

## Results

### An Immune Response Is Necessary for Endometriotic Lesion Formation

**Figure 1:**
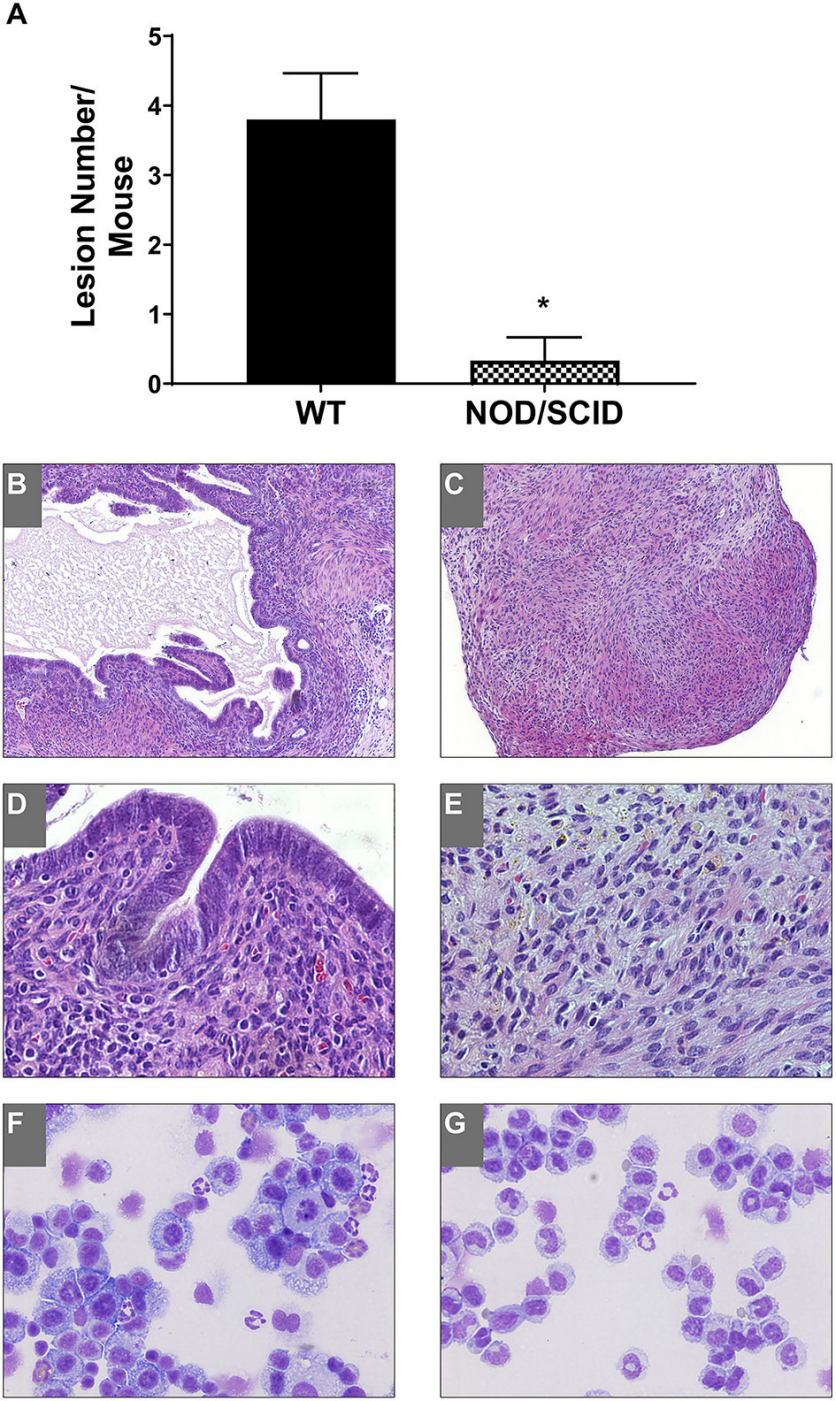
NOD/SCID to NOD/SCID transfers do not form endometriotic lesions and have decreased immune cells involved in the peritoneal cavity. **(A)** Endometriotic lesion numbers found in WT to WT and NOD/SCID to NOD/SCID uterine endometrial tissue transfer. **(B–E)** Histological evaluation (H&E) of lesion tissue. **(B,D)** WT to WT lesions. **(C,E)** NOD/SCID to NOD/SCID lesions. **(F,G)** Peritoneal lavage differentials. Magnification: 10×: **(B,C)** and 40×: **(D,E)**. *p < 0.05, one-tailed, unpaired *t*-test, Welsh's correction. WT, wild type.

To access the immune dependence of disease, NOD/SCID mice were used; these animals were severely immunocompromised with defects in natural killer dysfunction, low cytokine production, B/T cell dysfunction, absent complement, and defective macrophage and dendritic cell responses (Shultz et al., 1995). Uterine endometrial tissue was transferred from WT to WT and NOD/SCID to NOD/SCID to observe lesion formation. As we previously showed, E_2_ does not significantly affect lesion number; therefore, hormonally intact mice were used. Three weeks following uterine tissue transfer, tissue or attached lesions were removed from the mice. In WT to WT uterine tissue transfers, endometriotic lesions formed as expected and previously described (Burns et al., 2012); however, in the NOD/SCID to NOD/SCID uterine tissue transfers, few attached tissues were found (Figure 1A). Each piece of uterine tissue or lesion removed was histologically evaluated to characterize the tissue as a lesion: the presence or absence of stroma, epithelial lined glands, and/or hemosiderin-laden macrophages. Figure 1A shows a significantly decreased presence of lesion formation in the NOD/SCID to NOD/SCID uterine tissue transfer model. Histologically, as compared with the WT to WT lesions, the lesions/uterine tissues removed from the NOD/SCID to NOD/SCID were disorganized, lacked epithelial cells, and glandular structure, but hemosiderin-laden macrophages were present (Figures 1B–E). When examining differentials of peritoneal lavage, as expected, the WT to WT lavage contained neutrophils, macrophages, monocytes, and B/T cells (Figure 1F). In contrast to WT to WT peritoneal lavage, the differentials of the peritoneal lavage from the NOD/SCID to NOD/SCID (Figure 1G) had limited neutrophils that were immature, few to no macrophages, no B/T cells, but had monocytes. Together, these data further suggest that neutrophils and macrophages are key effector cells in the initiation of endometriotic lesion development.

### Uterine Tissue Is Necessary for Endometriotic Lesion Formation

**Figure 2:**
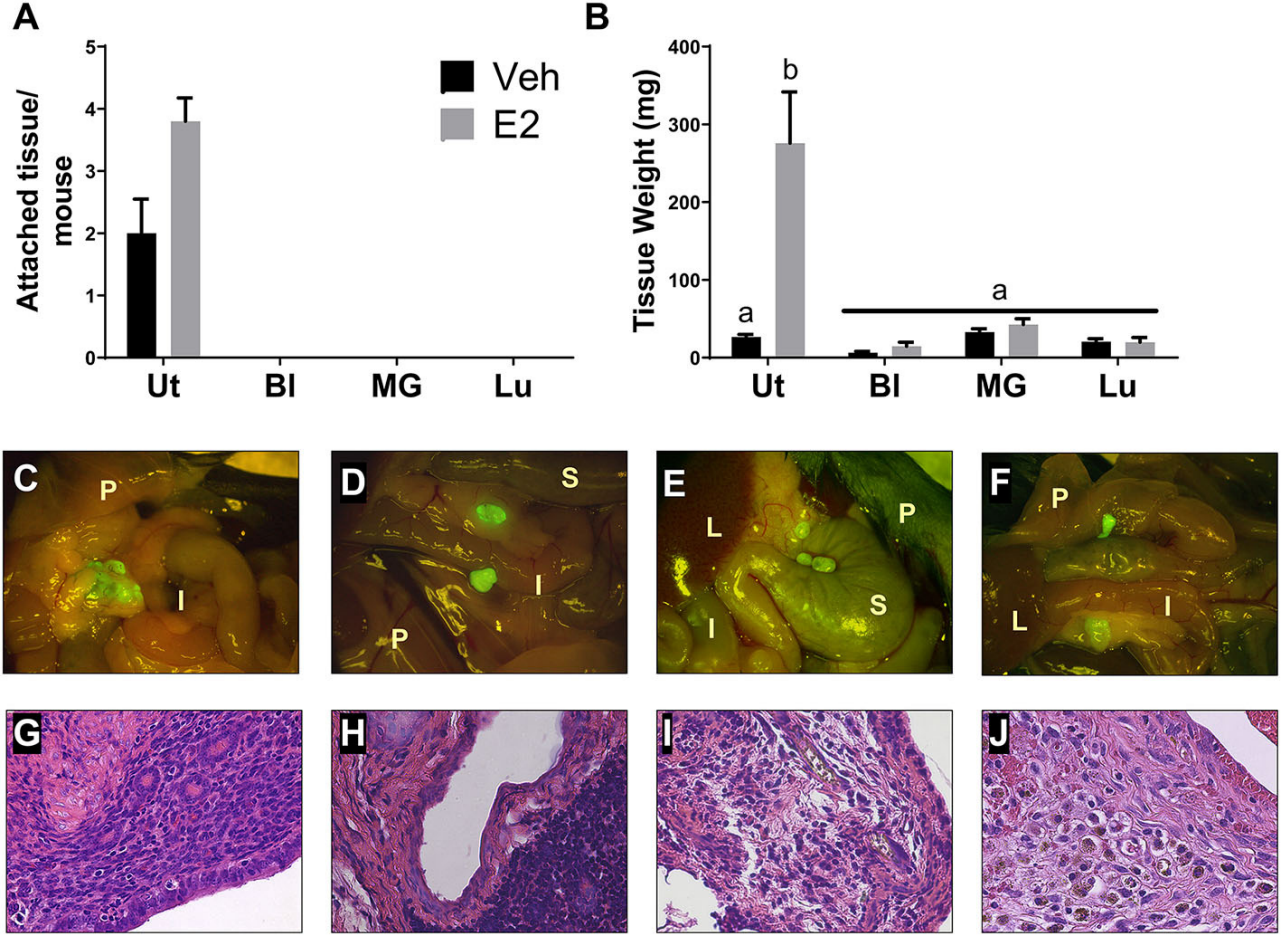
Endometriotic lesions are only formed when uterine tissue is dispersed into the peritoneal cavity. **(A)** Attached tissue number per mouse. Animals were treated with corn oil vehicle (Veh) or E_2_ valerate (2.5 μg/mouse/week) for 3 weeks. **(B)** Weight of tissue removed from the peritoneal cavity 3 weeks after tissue transfer. **(C–F)** Gross appearance of tissues (7.5×) with WT uterus **(C)**, bladder **(D)**, mammary gland **(E)**, or lung **(F)** tissue transfers 3 weeks after initiation. Lesions in C are attached to sites within the peritoneal cavity. Tissues in D–F are free floating throughout the peritoneal cavity and collected together for documentation. I, intestine; P, peritoneal wall; L, liver; S, stomach. **(G–J)** Histological evaluation (H&E) of lesion/unattached tissues (×400) with WT uterus **(G)**, bladder **(H)**, mammary gland **(I)**, or lung **(J)** tissue transfers 3 weeks after initiation. Representative examples are E_2_ treated. Letters different than each other are statistically significant, *p* < 0.05, Two-way ANOVA, Tukey's multi-comparison test. Ut, uterus; Bl, bladder; MG, mammary gland; Lu, lung.

To determine if endometriotic lesion formation is uterine dependent and not solely due to an immune response in the peritoneal cavity, WT tissue from organs having similar structure to the uterus (i.e. epithelial cells, stromal compartment, and outer muscle layer) and differing ESR1 and ESR2 levels were used. At necropsy, GFP positive uterine tissue was attached with 3-4 endometriotic lesions per mouse (Figures 2A,C). As expected, treatment with E2 did not significantly change lesion number in the uterine tissue transfers, but increased weight (Figure 2B). In contrast, no attached tissues/lesions were present in the animals that received bladder, mammary gland, or lung tissues (Figure 2A). The GFP positive tissue recovered from the bladder, mammary gland, and lung tissue transfers were not attached, were free floating in the peritoneal cavity, and were cream to white in color (Figures 2D–F; floating tissue was moved to similar spots for imaging). The amount of tissue removed from the bladder, mammary gland, and lung tissue transfers suggests that a portion of the tissue was phagocytized as the remaining tissue removed was less than the amount of tissue dispersed into the peritoneal cavity (Figures 2B,D–F). These data suggest that the presence of uterine selective factors is important in lesion development.

Lesions and tissue fragments removed from the peritoneal cavity were histologically examined for glandular structures, organization, and appearance. The uterine tissue injected into the peritoneal cavity, as previously published (Burns et al., 2012, 2017; Jones et al., 2018), forms endometriosis-like lesions that contain stroma, epithelial lined glands, and hemosiderin-laden macrophages (Figure 2G). When histologically examining the tissue fragments removed from the bladder, mammary gland, and lung tissue transfers, H&E staining revealed disorganized, fibrotic tissue having no glandular structures that contained immune cell infiltrations (Figures 2H–J).

**Figure 3:**
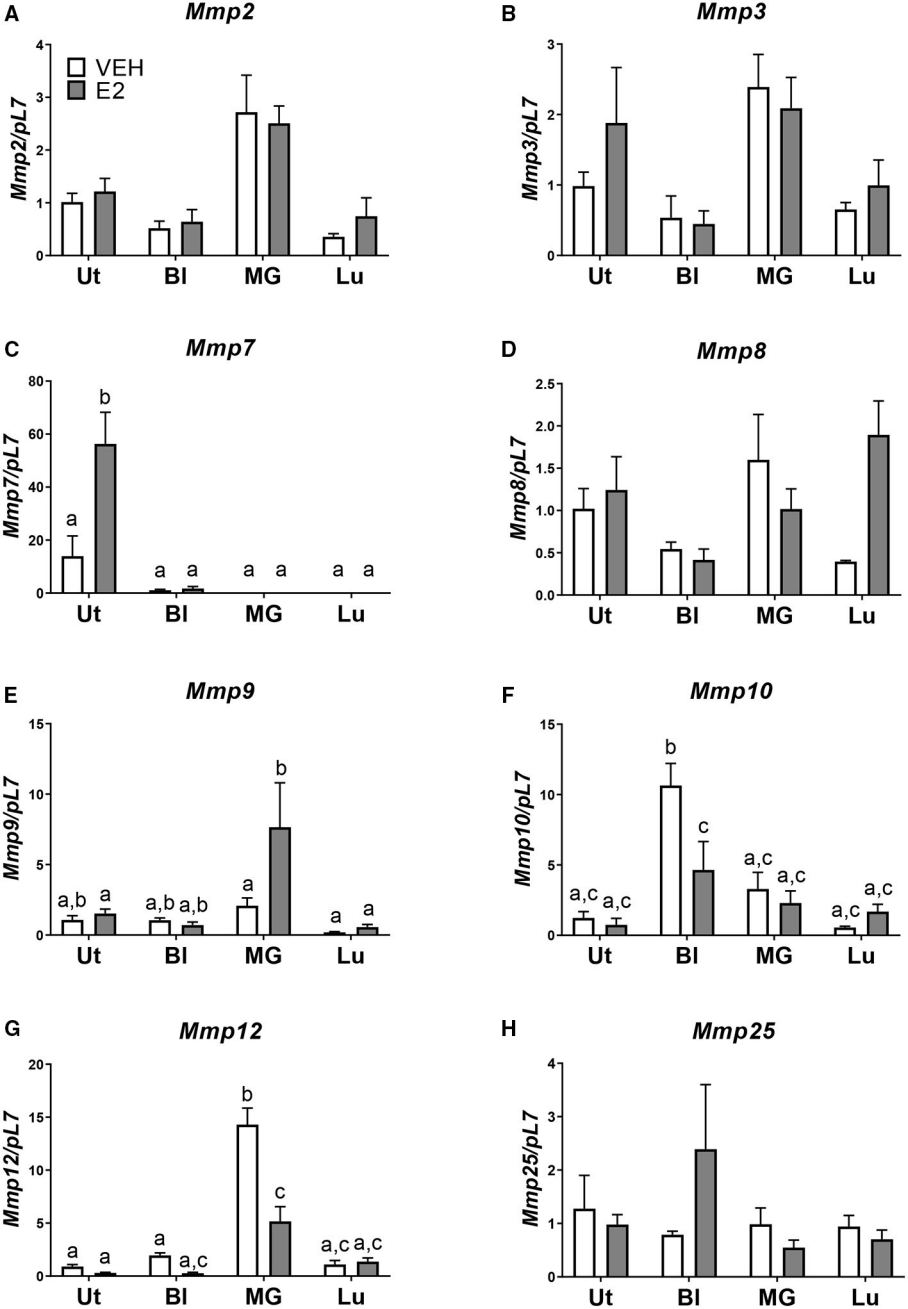
*Mmp7* is highly expressed in endometriotic lesions and enhanced with E_2_. **(A–H)** Gene expression of MMP family members in lesions/removed tissues. **(A)** *Mmp2*, **(B)** *Mmp3*, **(C)** *Mmp7*, **(D)** *Mmp8*, **(E)** *Mmp9*, **(F)** *Mmp10*, **(G)** *Mmp12*, and **(H)** *Mmp25*. Samples are normalized to uterus vehicle (set to 1) except for *Mmp7* in which bladder vehicle is set to 1. Tissue was removed, RNA was isolated, and gene expression was determined by RT-PCR. Letters different than each other are statistically significant, *p* < 0.05, two-way ANOVA, Tukey's post-test.

Next, we examined, at a basal level, what may be inherently different in the uterus from the bladder, mammary gland, and lung that may allow for the development of endometriotic lesions. Using the National Center for Biotechnology Information Gene Expression Omnibus (NCBI GEO) database, normal basal gene expression profiles of the uterus, bladder, mammary gland, and lung were mined for comparisons and contrasts. Stark differences in the metalloproteinase (MMP) family members were found. The expression levels of *Mmp2*, -*3*, -*7*, -*8*, -*9*, -*10*, -*12*, and -*25* were examined in the tissue removed from each tissue transfer. No statistical differences in *Mmp3*, -*8*, and -*25* levels were found among the uterus, bladder, mammary gland, and lung when normalized to uterus vehicle (Figures 3B,D,H). *Mmp2* showed increased expression in the mammary gland compared with the uterus vehicle (Figure 3A). *Mmp9* showed an increase in the mammary gland tissue after E_2_ treatment when normalized to uterus vehicle (Figure 3E). *Mmp10* showed an increase in the bladder tissue at the basal level that was repressed using E_2_ when normalized to the uterus vehicle (Figure 3F). The mammary gland had increased levels of *Mmp12* that significantly decreased using E_2_ treatment (Figure 3G). Finally, *Mmp7* showed uterine-derived lesions express high levels of *Mmp7* in comparison to the non-uterine–derived tissue, and this increase was accentuated in the presence of E_2_ (Figure 3C). Of note, the uterine lesion expression of *Mmp7* was normalized to bladder vehicle as the expression levels were higher than the other tissue types. Comparing vehicle treated uterine derived lesions to vehicle treated bladder, lung, or mammary gland removed tissue, a 13-fold increase, 52-fold increase, and a 528-fold increase of *Mmp7* was found, respectively. Likewise, in the presence of hormone, uterine derived lesion expression was increased 51 times compared to bladder removed tissue, 729 times compared to lung removed tissue, and 2029 times compared to mammary gland removed tissue. Together, these findings suggest that MMP7 may be an important factor for the invasion of uterine tissue into the organs of the peritoneal cavity to form endometriotic lesions.

### Blocking MMPs Halt the Invasion and Migration of Endometriotic Lesion Cells

**Figure 4:**
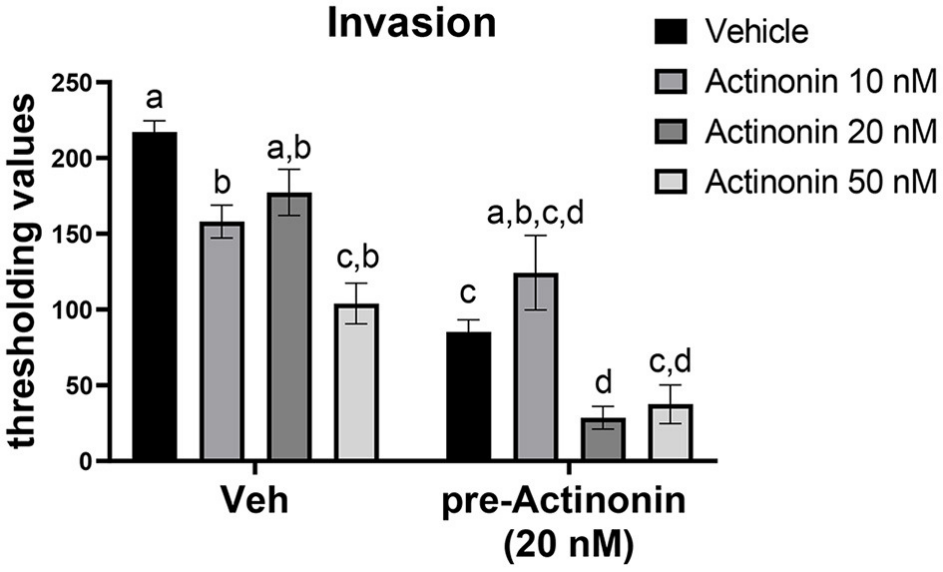
Blocking matrix metalloproteinases inhibits the invasion of mEmLe cells *in vitro*. mEmLe cell invasion through Matrigel (0.5 mg/ml) with vehicle (Veh) and actinonin treatment (10, 20, 50 nM) or 16 h pretreatment with actinonin (50 nM). Cells were allowed to invade for 16 h. Letters different than each other are statistically significant, *p* < 0.05, One-way ANOVA, Welsh's correction, Dunnett's T3 multi-comparison test.

To examine if inhibiting MMP7 would prevent invasion of endometriotic cells, we first generated a mEmLe cell line. The mEmLe cells spontaneously immortalized and expressed markers indicative of epithelial to mesenchymal transition (EMT) found in human endometriotic lesions [e.g., *Ctnnb1, Pgr, Esr1, Ptgs2, Mmp3, Tgfb1*, and *Krt18* (epithelial-derived marker that does not change with EMT) and are *CD44, CD45, F4/80* negative], similar to the lines published by Zeitvogel et al. (2001) without transformation from external factors (i.e., SV40 T-antigen). Actinonin is an antibiotic that broadly inhibits MMPs at differing concentrations: MMP1 (300 nM), MMP2 (5 nM), MMP3 (1,700 nM), MMP7 (19 nM) MMP8 (160 nM), MMP9 (330 nM), MMP10 (22 nM), MMP12 (4 nM), and MMP13 (13 nM). Therefore, to examine the effect of blocking MMP7, 10, 20, and 50 nM concentrations were chosen. These concentrations would inhibit MMP2, MMP7, MMP10, MMP12, and MMP13, but since MMP2, MMP10, and MMP12 are at low levels in the uterine lesions, actinonin would target MMP7 in the cells at this concentration. Treatment with actinonin significantly inhibited the invasion of the mEmLe cells through Matrigel (Figure 4). When cells were pretreated with actinonin (50 nM) and then treated with 10, 20, or 50 nM of actinonin, invasion was further inhibited (Figure 4). These data suggest that MMP7 in uterine tissue may play a role in the invasion and maintenance of endometriotic lesions.

## Discussion

To understand the etiology of endometriosis, we used a mouse model of disease in which minced donor tissue is dispersed into the peritoneal cavity of a host mouse. In theory, as mice do not menstruate and have a closed reproductive system, this method mimics the presence of retrograde tissue in the peritoneal cavity.

We previously used this model to examine the role of ESR1 and ESR2, E2, and the innate immune system in disease (Burns et al., 2012, 2017). We find this model mimics the establishment of disease by attaching naturally in the peritoneal cavity to sites mimicking human disease (e.g. intestine, cul-de-sac, intestinal mesentery, uterine blood supply, diaphragm, etc.), by traversing stages of lesion development from hemorrhagic, to patterning, to cystic, to deep infiltrating; however, this model does not mimic the development of endometriomas as the ovaries in mice are covered by a bursa (Burns et al., 2012, 2017; Jones et al., 2018). To expand on these findings, in this study, we first examined the role of the immune system in endometriotic lesion development. NOD/SCID mice were chosen as they are severely immunocompromised (Shultz et al., 1995) in contrast to the Rag-1/γ(c) knockout mice (Goldman et al., 1998), e.g., that are deficient in B/T cell populations and accept the implantation of human endometriotic lesions. In multiple studies using NOD/SCID mice as hosts to human endometriotic lesions, the tissue is sewn into the peritoneal cavity, implanted subcutaneously, placed under the kidney capsule, and/or lesion histology is not shown (Zamah et al., 1984; Bergqvist et al., 1985a,b; Aoki et al., 1994; Bruner et al., 1997; Awwad et al., 1999; Nisolle et al., 2000; Bruner-Tran, K. L. et al., 2002b; Hull et al., 2003; Greenberg and Slayden, 2004; Unno et al., 2014). In our study, the use of the NOD/SCID mice helps to confirm that the initiation of endometriosis has a strong immunological component to disease development. Histological association of the lesion/uterine tissue removed from the NOD/SCID to NOD/SCID suggests that the required effector cells and/or signaling processes are absent for lesions to develop glands, stroma, epithelial lined glands, and hemosiderin-laden macrophages. The use of this model further suggests that the innate immune system is important in disease development as the Rag-1/γ(c) knockout mice (adaptive; B/T cell deficient) do not have a defect in the development of lesions from transplanted tissue (Greenberg and Slayden, 2004; Bruner-Tran et al., 2010). Our ongoing and further studies are directed at the careful examination of the innate immune cell types and the innate immune cell activation states.

A common question surrounding the etiology of endometriosis and the development of endometriosis-like disease in a mouse model pertains to the initiation of the disease being an immune response where any tissue injected into the peritoneal cavity would form lesions. In this study, to determine if endometriotic lesion development is a uterine-specific disease, the uterus, bladder, mammary gland, and lung tissue were minced and dispersed into the peritoneal cavity. The non-uterine tissues express ESR1 and ESR2, albeit at different levels (Stefan and Kenneth, 2001), and have similar cellular composition (i.e. epithelial, stoma, and muscle layers); therefore, these factors could also be addressed in disease development. Our findings demonstrate that only uterine tissue attached and formed lesions, while the non-uterine tissues were found floating as small pieces throughout the peritoneal cavity with no attachments. Interestingly, when attempting to search for other peritoneal adhesive diseases that might form as a result of lesions in the cavity, no other diseases of this kind were found. Together, these findings further suggest that endometriosis is a uterine-specific disease.

Tissue remodeling of the uterus is regulated by a balance of MMPs and their inhibitors that are often hormonally regulated (Balkowiec et al., 2018). The interplay between these factors is important for cyclic preparation of the endometrium for embryo implantation, endometrial shedding, and endometrial renewal (Balkowiec et al., 2018). Aberrant regulation of MMPs is implicated in the development of endometriosis (Bruner et al., 1997, 1999; Bruner-Tran et al., 2002a, 2006; Fazleabas et al., 2002; Osteen et al., 2002, 2005; Ueda et al., 2002; Gilabert-Estelles et al., 2003, 2007; Matsuzaki et al., 2010; Liu et al., 2015; Szymanowski et al., 2016; Zhao et al., 2017; Logan et al., 2018). Thus, upon examining the basal transcription levels of genes expressed in the bladder, mammary gland, and lung tissues compared to the uterine tissue, finding differences in *Mmps* was not fully unexpected. Based on these findings, we explored various *Mmp* candidates in the tissues removed from the peritoneal cavity of the host mice after uterine, bladder, mammary gland, and lung tissue transfers. What was surprising is only *Mmp7* was dramatically more expressed in the endometriotic lesions than the other *Mmps* and that this transcript was further increased with E_2_ treatment.

MMP7 is a cell adhesion extracellular matrix remodeling and matrix MMP that is expressed upon β-catenin activation and is involved in the mediation of epithelial to mesenchymal transition *via* FasL (Ke et al., 2017). It is detected in the epithelial layer of the uterus (Rodgers et al., 1993) with extracellular matrix components such as collagen IV, E-cadherin, fibronectin, laminin, Fas-ligand, and β4 integrin, as substrates (Balkowiec et al., 2018). In the mouse uterus, MMP7 is epithelial and also colocalizes with neutrophils during re-epithelization of the new epithelium (Kaituu et al., 2005). Furthermore, in a mouse model of decidualization and menstruation, the Salamonsen Laboratory additionally shows that MMP3 and MMP7 were abundant during re-epithelization in close proximity to newly reforming epithelium (Kaituu et al., 2005). In this model, once progesterone is withdrawn, the decidualized endometrium entirely degrades and sheds in 24 h, but equally as quickly, re-epithelization precedes stromal restoration with the repair being complete by 48 h after progesterone withdrawal. Similarly, in the human uterus, MMP7 is found in the epithelial layer and is expressed at low levels during the proliferative, secretory, and menstrual phases of menstruation (Rodgers et al., 1994; Matsuzaki et al., 2010). This is in contrast to significantly higher levels of MMP7 found in all phases of the menstrual cycle in uterine biopsies of women with deep infiltrating endometriotic lesions (Matsuzaki et al., 2010). Furthermore, in ectopic endometriotic lesions, Matsuzaki et al. (2010) showed that red endometriotic lesions have much higher levels of MMP7 than deep infiltrating lesions, ovarian endometriomas, and black peritoneal lesions. Endometriotic lesions can be staged (Nisolle et al., 2000; Fazleabas et al., 2002) with the red lesions being newly developing and most active; thus, the need for invasion and the invasive properties of MMP7 may be implicated in the development of endometriotic lesions. Additionally, supporting human findings, based upon our previous data of lesions at 3 and 6 weeks, lesions removed at 6 weeks express lower Mmp7 compared to lesions removed at 3 weeks (Burns et al., 2012; Jones et al., 2018).

In our study, we blocked the activity of MMP7 with actinonin, a broad-spectrum MMP inhibitor. At the concentrations used and based on the MMPs found in our uterine lesions, we primarily targeted MMP7, MMP10, MMP12, and MMP13. Therefore, we targeted MMP7 and MMP10, but MMP10 was minimally expressed in our uterine lesions to focus on the inhibition of MMP7. From these studies, we found that inhibiting and pre-blocking MMP7 in mEmLe cells significantly decreased the invasive properties of these cells. Together, our findings suggest that MMP7 likely plays a role in the ability of uterine tissue and the innate immune system to establish and maintain endometriotic lesions. Future studies will address the role of MMP7 in endometriotic lesion development and as a possible target for the development of a therapeutic treatment.

## Data Availability Statement

The original contributions presented in the study are included in the article/supplementary material, further inquiries can be directed to the corresponding author/s.

## Ethics Statement

The animal study was reviewed and approved by National Institutes of Health Guidelines for Humane Use and Care of Animals University of Cincinnati's Institutional Animal Care and Use Committee.

## Author Contributions

SM, KK, and KB contributed to the conception and design of the study. SM and KB contributed to the execution of the study. All authors contributed to manuscript revision, read, and approved the submitted version.

## Funding

This research was supported by the National Institutes of Health Grant Z01 ES70065 (KK) and R00 ES021737 and R01 HD097597 (KB).

## Conflict of Interest

The authors declare that the research was conducted in the absence of any commercial or financial relationships that could be construed as a potential conflict of interest.

## Publisher's Note

All claims expressed in this article are solely those of the authors and do not necessarily represent those of their affiliated organizations, or those of the publisher, the editors and the reviewers. Any product that may be evaluated in this article, or claim that may be made by its manufacturer, is not guaranteed or endorsed by the publisher.
